# Knowledge, Attitudes, and Practices of Mothers of Preschool Children About Oral Health in Qatar: A Cross-Sectional Survey

**DOI:** 10.3390/dj6040051

**Published:** 2018-10-01

**Authors:** Asmaa Alkhtib, Abdul Morawala

**Affiliations:** 1 Oral Health Division Operations, Primary Health Care Corporation, Doha 26555, Qatar; drasmaaalkhtib@gmail.com; 2 Department of Dentistry, Primary Healthcare Corporation, Specialist Pediatric Dentist, Doha 26555, Qatar

**Keywords:** dental caries, knowledge, mother, oral health, preschool children, Qatar

## Abstract

Health-related behaviors are influenced by knowledge and awareness, with oral health being no exception. It is well-known that oral diseases are influenced by social determinants. There is an association between the oral health knowledge of mothers and the status of their children’s oral health. In Qatar, the knowledge and practices of oral health in preschool children have not been previously reported. The aim of this study was to assess the knowledge, attitude, and related practices of mothers of preschool children about oral health in Qatar. A total of 400 questionnaires were distributed by the principals of kindergarten to mothers of children attending 16 government kindergartens in Qatar. The questionnaire included 38 close-ended questions grouped into nine categories, addressing different aspects of knowledge and practices related to early childhood oral health. The questionnaire was constructed in English, before being translated into Arabic, which is the local language in Qatar. The questionnaire instrument was pre-tested on mothers with demographic characteristics matching the main population. These participants were not included in the main study. The questionnaire study was associated with a clinical epidemiological study to assess dental caries and enamel defects of the sampled children. The dmft caries index (decayed, missing and filled teeth) was used for that purpose according to the World Health Organization criteria. For the questionnaire administered to mothers with clinical survey variables, a binary logistic regression analysis was performed to determine the associations between the measures of oral health status (dmft, Dental index) and mothers’ oral health knowledge and practices. A total of 48% mothers thought that children should have their teeth brushed from the age of three years and 42% chose younger than two years as a starting age for brushing. More than half (54%) of the mothers thought that children should not have their teeth flossed. In general, no significant statistical association was found between dmft and any other variables, except for whether or not the child had visited the dentist. Logistic regression analyses were performed to determine the association between the measures of oral health status (dmft, DI) and mothers’ oral health knowledge and practices. After controlling for the other independent variables included in this model, the test of the model was not statistically significant, which indicated that none of the variables represent a significant risk for occurrence of caries. The only exception was whether or not the child had visited the dentist (odds ratio = 2.51, 95% confidence interval 1.091–5.774). Despite the existence of good knowledge of oral health care, there were deficiencies in the oral health care provided to children. This may reflect that seeking dental care is either not very important or it is challenging to obtain access to a child-friendly dentist in the public health system in Qatar. The results of this study suggest that there is a need for an oral health promotion program to fill the gaps in knowledge for mothers regarding oral health care for young children.

## 1. Introduction

Health-related behaviors are influenced by knowledge and awareness, with oral health being no exception. There is an association between oral health knowledge, age, and the education level of mothers, which are directly linked to the status of their children’s oral health [1,2,3,4]. Oral health is an integral component of general health that plays an essential role in the life of a child. Dental caries are one of the pertinent oral health problems that are universally present. In most developing countries, the levels of dental caries are steadily rising. Countries in the Middle East have demonstrated a high prevalence of early childhood caries (ECC) [5,6]. A recent study in Qatar showed that the prevalence of ECC is 89% and the dmft caries index (decayed, missing and filled teeth) is 7.6.

In Qatar, the oral health knowledge and practices of mothers in relation to the oral health of their children have not been reported previously. Hence, the aim of the study was to assess the knowledge, attitude, and related practices of mothers of preschool children about oral health in Qatar.

## 2. Materials and Methods

Ethical clearance was obtained from the Human Ethics Research Committee at the University of Melbourne (#1034161) and the Medical Research Centre at Hamad Medical Corporation in Qatar (#10097). The Government of Qatar authorized this study involving pre-school children aged 3–4 years in the public kindergartens. A questionnaire assessing the knowledge and attitude of mothers toward the oral health of preschool children was developed based upon the primary researcher’s knowledge as a pediatric dentist [7]. The questionnaire included 38 close-ended questions grouped into 9 categories, addressing different aspects of knowledge and practices related to early childhood oral health. The questionnaire was constructed in English before being translated into Arabic, which is the local language in Qatar. The questionnaire instrument was pre-tested on a sample of 7 mothers with demographic characteristics similar to those of the test population. These participants were excluded from the main study. The sample for this study aimed to be representative of children attending governmental kindergartens in Qatar. The total number of children in governmental kindergartens in the school year of 2011–2012 was 6374, so the sampled children represented about 4% of the eligible population at the time of the study.

The questionnaire was part of an information package that the family received from the kindergarten principals. Written consent was obtained from all participants. Dental caries of the children were also recorded using the dmft index according to the World Health Organization criteria (1997) [8]. The questionnaire data were entered into Microsoft^®^ Excel 2003 spreadsheets (Microsoft Corporation, Seattle, WA, USA) and exported to SPSS 20.0™ for Windows^®^ (SPSS, Chicago, IL, USA). Inferential statistics were used to examine any possible associations between dependent variables and independent variables. The outcome measures (dependent variables) included the oral health knowledge of mothers in general and the knowledge pertaining to their children’s oral health, the oral health practices of mothers and those provided to their children, and the dietary habits of the children. The independent variables were early feeding habits, dietary habits, oral hygiene practices, and oral health care provided to children and mothers.

For the questionnaire administered to mothers and the clinical survey variables, a binary logistic regression analysis was performed to determine the associations between the measures of oral health status (dmft, Dental Index) and mothers’ oral health knowledge and practices. Such analyses were used to assess the association between the potential risk factors and the development of caries, which may assist in developing a prediction model. All explanatory variables in the set within the model (the so-called “enter method”) were fitted into a single model, where each variable was considered a potential confounder and the data were analyzed with and without controlling for the potential confounder. Results with *p*-values less than 0.05 were considered statistically significant.

## 3. Results

Most mothers returned the questionnaires, as there was a high response rate of 316/400 (79%). Table 1 shows the demographic data of the study participants.

The majority of the children were breast-fed (71%) and bottle-fed (83%). Interestingly, 24% of the children were never breast-fed at all. Thirty-six percent of the children went to sleep with a milk bottle. The most common (40%) content of the bottle was cow’s milk. Around 37% of mothers provided on-demand breast-feeding and 30% provided on-demand bottle-feeding. When mothers were asked to rate several types of food and snacks for their potential effect on teeth, their overall knowledge was reasonable. Most mothers (63–90%) knew that all types of sweets (sugar, candies, and chocolate), retentive carbohydrates (potato chips), and soft drinks were bad for teeth and that healthy food items (vegetables, fruits, milk, and cheese) were good for teeth. However, there was less than optimal knowledge about the potential harmful effects of orange juice on teeth, as 85% of mothers thought it was good for the teeth (Table 2).

More than half (61%) of the mothers reported that they had tooth decay or gum problems. In terms of visiting the dentist, only 38% reported that they would go every six months and 18% would go every year. A striking finding was that 25% of mothers did not remember “when was the last time you went to the dentist?” despite most (78%) of the mothers having stated that they brushed their teeth twice per day. A total of 48% thought that children should have their teeth brushed from the age of three years and 42% chose younger than two years as a starting age for brushing. More than half (54%) of the mothers thought that children should not have their teeth flossed (Table 3).

Half of the children had not yet visited the dentist, and of those who did (43%), most (61%) of their mothers did not answer the question about when the child first had visited the dentist. Of the children who visited the dentist, only 10% went for a regular checkup. More children visited the dentist when a problem occurred, such as having a toothache (14%) or having a cavity and needing a filling (16%). The mean age of commencing tooth brushing was three (±0.9) years. More than half (53%) of the children brushed their teeth by themselves and 48% had parental assistance in brushing. There were 248 cases for which the results from both the clinical examination and the mother’s questionnaire were available. This allowed us to examine the association between the survey results and the dental caries experience. In general, no significant statistical association was found between dmft and any other variables, except for whether or not the child had visited the dentist. Table 4 shows the association between the knowledge of the mother and the frequency of caries of the child based on the dmft index.

Logistic regression analyses were performed to determine the association between the measures of oral health status (dmft, DI) and mothers’ oral health knowledge and practices. After controlling for the other independent variables included in this model, the test of the model was not statistically significant, which indicated that none of the variables represent a significant risk for occurrence of caries, except for whether or not the child had visited the dentist (odds ratio (OR) = 2.51, 95% confidence interval (CI) 1.091–5.774).

## 4. Discussion

Parental knowledge, attitude, and practices can have an impact on children’s oral health. Children under the age of five years generally spend most of their time with parents and guardians. These early years involve “primary socialization”, during which the earliest childhood routines and habits are acquired [9,10]. During the first three years during the pre-school period, the role of parents is important in maintaining the good oral health of the child [11]. The present study findings focused on mothers’ knowledge, attitudes, and practices toward the oral health of preschool children in Qatar that have not previously been reported. These results are comparable to other results conducted in similar cohorts of mothers in the USA [12] and Saudi Arabia [13,14,15]. In the current study, the findings highlighted some practices that are considered to increase the risk of dental caries. Around one-third (36%) of the children went to bed with a bottle that mostly contained milk (40%). There was no differentiation in the questionnaire between formula or cow’s milk, which is the most commonly used milk in Qatar. Over one-third (42%) of the children were reported to snack frequently and the preferred snacks were mostly cariogenic. Frequent consumption of sweetened drinks and starchy food (cariogenic diet) has been found to be associated with the occurrence of tooth decay in several studies [16,17,18]. In a study carried out in Nepal, only 29% of the respondents had knowledge that prolonged and frequent bottle-feeding affects dental health [19]. The results of this study were similar to the results of studies conducted by Moulana et al. [20], Wyne et al. [21], Kamolmatyakul and Saiong [22], and Chan et al. [23], in which the majority of the mothers were aware that consumption of sugary items can lead to dental caries. However, there was little awareness about the different forms of sugary items, apart from chocolates, that are harmful to the teeth. This has shed light on inadequate knowledge about the relationship between the different forms of sugar consumption and dental caries. All these findings are suggestive of poor knowledge about oral health and indicates the need for effective oral health education programs.

Several independent studies and the American Academy of Pediatric Dentistry have recommended early dental visits for children, which should ideally take place before one year of age or within six months of the eruption of primary teeth. This is strongly supported by the American Dental Association [24,25,26,27,28]. The study by Chabra et al. found that only 15.2% of the parents were aware that the first dental visit of the child should occur before the age of one [29], whereas Hussein et al. reported even lower awareness among parents (12.5%) in terms of this knowledge [30].

Many mothers (61%) had oral health problems and a quarter of them could not remember the last time they visited the dentist. This may provide an indication that seeking dental care is not a priority for these mothers or there might be difficulties in accessing dental care despite dental services in Qatar being readily available in each suburb. Despite the good knowledge about oral health care, there was a significant deficiency in the oral health care and oral hygiene provided to children. Many mothers did not answer critical questions about the dental care provided to their child: 61% did not answer the question “When did your child visit the dentist?”, 59% did not answer “Why did your child visit the dentist?”, and 57% did not answer “What type of treatment was provided to the child when the child visited the dentist?”. This may reflect that seeking dental care is either not important for them or it is challenging to obtain access to a child-friendly dentist in the public health system in Qatar.

In our study, we observed that although knowledge of the various aspects of oral health and dental decay was present, the attitudes of a number of the mothers toward oral health practices were found to be unhealthy and would set a bad precedent for the growing child. This indicates that they were unable to translate their knowledge into habit. This needs to be further discussed and scrutinized. The literacy rate among Qatari women is 97.6%. Although the majority of mothers had sound knowledge about oral health in children, low motivation, low enthusiasm, and the lack of practical training could be a result of a poor implementation of knowledge [31,32,33,34]. Parents should be considered key persons in ensuring the well-being of young children. In addition, appreciating their knowledge, attitude, and practices about their children’s oral health may help the dental community to understand some of the reasons why children do not receive the dental care that they need [32].

This study had limitations, including sampling bias. The sample was obtained exclusively from government kindergartens, which cater only to Qatari children. Qatari nationals represent about 20% of the population and the remaining 80% are from Arabic and non-Arabic ex-patriates. If private kindergartens were included in the sampling frame and non-Qatari children were sampled, the results may have been different. The general cultural perception about Qatari children is that they are spoiled in many ways and indulging their diet is one of them. Therefore, these results may represent the “tip of the iceberg”. Furthermore, in this study, children who did not attend kindergartens were not included in the sample. Mothers of children who do not attend kindergarten may have different views from those who send their children to kindergarten. Another bias is the non-response bias: the 21% of mothers that did not respond may have had different perspectives than those who responded to the questionnaire. Finally, the self-reported responses might not represent mothers’ true knowledge and behaviors. Mothers may report what they think should be the correct practice or knowledge rather than the truth. The statistically significant association between the occurrence of caries and whether the child visited the dentist might be a random finding or may reflect the fact that only children with dental caries visit the dentist.

## 5. Conclusions

The results of the questionnaire reflected that the oral health knowledge of mothers is reasonable, although there is room for improvement in oral health messages on the starting age for brushing and the importance of flossing. The general dietary knowledge of mothers was good, except for their knowledge about the harmful effects of orange juice on teeth. Despite the good knowledge about oral health care, there was a significant deficiency in the oral health care and oral hygiene provided to children. This also reinstates the urgent need to plan and conduct appropriate oral health programs targeting the two different groups through strategies that are tailored to their understanding and requirements. More emphasis should be placed on improving their level of knowledge, which would ultimately be reflected in their oral health behavior. Health education should focus on parental responsibility for oral health and mothers should be encouraged to provide practical and emotional support to their children with regard to oral hygiene habits. This may reflect the fact that seeking dental care is either not important for them or it is challenging to obtain access to a child-friendly dentist in the public health system in Qatar. The results of this study suggest that there is a need for an oral health promotion program to fill the gaps in knowledge for mothers regarding oral health care for young children.

## Figures and Tables

**Table 1:** (**a**) Distribution of caries in Qatari children by gender (dmft) and (**b**) the demographic data of the participants.

(**a**)					
**Caries Experience Distribution:**	**Sex**		**Total *n* = 250 (%)**	**Mean (±SD)**	**Range**
	Female *n* (%) *	Male *n* (%) *			
Decayed teeth	110 (44)	113 (45)	223 (89)	7 (5.0)	0–20
Missing teeth	16 (6)	23 (9)	39 (15)	0.3 (0.8)	0–6
Filled teeth	18 (7))	19 (8)	37 (15)	0.3 (0.8)	0–7
dmft * (±SD)	7.6 (±5.3)	7.6 (±4.9)	-	7.6 (±5.0)	0–20
SiC * (±SD)	-	-	-	13.6 (±2.5)	
Caries prevalence	44%	45%	89%		
Caries free	13 (5)	14 (6)	27 (11)	-	-
Soft tissue (abscessed teeth)	9 (3)	14 (6)	23 (9)	-	-
(**b**)					
**Demographics**		**Number**		**Percentage**	
**Sex of children**					
Male		146		46	
Female		169		53	
Total		315		100	
**Nationality**					
Qatari		305		96	
Non Qatari		10		04	
Total		315		100	
Mother’s education level (*n* = 298) *					
Primary level		23		7	
High school		145		46	
University		130		41	
Age group of mother (*n* = 298) *					
16–25 years		36		11	
26–34 years		181		57	

* Percentage calculated out of total participants.

**Table 2:** Distribution of the study participants based upon their dietary knowledge (*n* = 315).

Impact of Food Stuff on the Child’s Oral Health	Missing	Good	Bad	I Do Not Know
**Sweets**				
Sugar	20 (6)	24 (8)	254 (80)	18 (6)
Candies	18 (6)	9 (3)	284 (90)	5 (1)
Chocolates	18 (6)	14 (4)	277 (88)	7 (2)
Retentive carbohydrates (potato chips)	19 (6)	41 (13)	200 (63)	56 (18)
**Sweetened drinks**				
Soft drink	19 (6)	1 (0.3)	285 (90)	11 (3)
Orange juices	21 (7)	269 (85)	14 (5)	12 (3)
**Healthy foodstuff**				
Vegetables	17 (5)	296 (94)	1 (0.3)	2 (1)
Fruits	17 (5)	297 (94)	0	2
Milk	17 (5)	296 (94)	0	3
Cheese	20 (6)	294 (94)	0	2

**Table 3:** Knowledge of mothers related to the child’s oral health.

	Replied (%)	Missing (%)
**How often do you think people should see the dentist?**		14 (5)
When they have dental problem (e.g., toothache)	49 (16)	
Every 5 years	0	
Every 2 years	4 (1)	
Every year	33 (10)	
Every 6 months	216 (68)	
**At what age should children first be taken to the dentist?**		19 (6)
Older than 6 years of age	104 (33)	
At 6 years	56 (18)	
At 3 years	136 (43)	
Younger than 2 years	1 (0.3)	
**At what age should children have their teeth brushed?**		16 (5)
Older than 6 years of age	3 (1)	
At 6 years	12 (4)	
At 3 years	152 (48)	
Younger than 2 years	133 (42)	
**Do you think children should have their teeth flossed?**		26 (9)
No	172 (54)	
Yes	118 (37)	
**If yes, at what age children should floss their teeth?**		193 (61)
Older than 6 years of age	13 (4)	
At 6 years	44 (14)	
At 3 years	46 (15)	
Younger than 2 years	20 (6)	

**Table 4:** Association between caries experience of the child and mother’s knowledge.

Oral Health Knowledge and Practices of Mothers	No or Low Caries *n* (Valid %)	Severe Caries *n* (Valid %)	Sig. (2-Sided Exact Test)
**Mother’s age (*n* = 232)**			
16–34 years	48 (21)	119 (51)	0.133
35 years or older	12 (5)	53 (23)	
**Mothers’ education (*n* = 235)**			
Primary/high school	35 (15)	106 (45)	
University	26 (11)	68 (29)	0.651
**Has the child visited the dentist (*n* = 235)**			
No	43 (18)	87 (37)	
Yes	19 (8)	86 (36.6)	0.011 *
**Rate orange juice for effect on teeth (*n* = 234)**			
Good	60 (26)	154 (66)	
Bad/I don’t know	2 (1)	18 (8)	0.111
**Was the child bottle-fed (*n* = 235)**			
No	6 (3)	25 (11)	
Yes	56 (24)	148 (63)	0.390

* = statistically significant.
